# Mining the Selective Remodeling of DNA Methylation in Promoter Regions to Identify Robust Gene-Level Associations With Phenotype

**DOI:** 10.3389/fmolb.2021.597513

**Published:** 2021-03-26

**Authors:** Yuan Quan, Fengji Liang, Si-Min Deng, Yuexing Zhu, Ying Chen, Jianghui Xiong

**Affiliations:** ^1^ Hubei Key Laboratory of Agricultural Bioinformatics, College of Informatics, Huazhong Agricultural University, Wuhan, China; ^2^ Lab of Epigenetics and Advanced Health Technology, Space Science and Technology Institute, Shenzhen, China; ^3^ State Key Laboratory of Space Medicine Fundamentals and Application, China Astronaut Research and Training Center, Beijing, China; ^4^ Aromability Inc., Beijing, China; ^5^ Jiangsu Industrial Technology Research Institute (JITRI), Applied Adaptome Immunology Institute, Nanjing, Jiangsu, China

**Keywords:** DNA methylation, remodeling, gene level, SIMPO algorithm, phenotype-associated genes

## Abstract

Epigenetics is an essential biological frontier linking genetics to the environment, where DNA methylation is one of the most studied epigenetic events. In recent years, through the epigenome-wide association study (EWAS), researchers have identified thousands of phenotype-related methylation sites. However, the overlaps of identified phenotype-related DNA methylation sites between various studies are often quite small, and it might be due to the fact that methylation remodeling has a certain degree of randomness within the genome. Thus, the identification of robust gene-phenotype associations is crucial to interpreting pathogenesis. How to integrate the methylation values of different sites on the same gene and to mine the DNA methylation at the gene level remains a challenge. A recent study found that the DNA methylation difference of the gene body and promoter region has a strong correlation with gene expression. In this study, we proposed a Statistical difference of DNA Methylation between Promoter and Other Body Region (SIMPO) algorithm to extract DNA methylation values at the gene level. First, by choosing to smoke as an environmental exposure factor, our method led to significant improvements in gene overlaps (from 5 to 17%) between different datasets. In addition, the biological significance of phenotype-related genes identified by SIMPO algorithm is comparable to that of the traditional probe-based methods. Then, we selected two disease contents (e.g., insulin resistance and Parkinson’s disease) to show that the biological efficiency of disease-related gene identification increased from 15.43 to 44.44% (*p*-value = 1.20e–28). In summary, our results declare that mining the selective remodeling of DNA methylation in promoter regions can identify robust gene-level associations with phenotype, and the characteristic remodeling of a given gene’s promoter region can reflect the essence of disease.

## Introduction

Epigenetics is a branch of genetics that studies the heritable changes in gene expression without changing the nucleotide sequence of a gene (Fraga et al., 2005), including DNA methylation, histone modification, and regulation of noncoding RNA, among which DNA methylation is one of the focuses in epigenetics (Dahl and Guldberg, 2003). Several studies have shown that the regulation of genes by DNA methylation is associated with the occurrence and development of various diseases, such as cancer (Jones, 2012; Akhavan-Niaki and Samadani, 2013; Mikeska and Craig, 2014), cardiovascular and cerebrovascular diseases (Kim et al., 2010; Peng et al., 2014; Wise and Charchar, 2016), and metabolic diseases (Cooper and El-Osta, 2010; Simar et al., 2014).

Similar to GWAS (genome-wide association study), EWAS can compare variations between patients and healthy people at the DNA methylation level and associate epigenetic variations with complex diseases as well as interpret the pathogenesis of complex diseases at the epigenetic level (Flanagan, 2015). EWAS open the door to study complex diseases, allowing researchers to find several previously undiscovered disease-related methylation sites, providing more epigenetic mechanisms for the pathogenesis of complex diseases (Li et al., 2019b; Liu et al., 2019). Since 2009, when the first EWAS was published, EWAS research has grown exponentially in recent years, reaching 618 publications in 2019 (Li et al., 2019b). Due to the availability of whole blood DNA methylation data, the experimental materials of most current EWAS studies are focused on whole blood tissues (Li et al., 2019b).

In the detection of clinical samples, the human DNA methylation chip is a common method for high-throughput EWAS analysis. The current widely used methylation chip is the Illumina 450 K BeadChip (Flanagan, 2015; Li et al., 2019b; Liu et al., 2019). However, multiple methylation probes are distributed in the same functional region of the same gene in the 450 K BeadChip, and different probes will be detected with different methylation values. In addition, because the methylation modification has a certain degree of randomness on the genome, the results of similar EWAS studies are often inconsistent (Xu et al., 2018). For example, there are several EWASs that focus on smoking-related phenotypes and identify tens of thousands of significantly different probes (Zeilinger et al., 2013; Dogan et al., 2014; Guida et al., 2015; Joehanes et al., 2016; Lee et al., 2016; Jenkins et al., 2017; Marabita et al., 2017; Zhang et al., 2018). We found that starting from these different probes, each independent EWAS can correspond to 101∼6,180 differential genes, and these EWAS publications predicted a total of 7,340 genes. However, only 1,334 (18.17%) of these genes were present in two or more independent EWASs. In addition, four diabetes-related EWAS projects had predicted 493 (Kang et al., 2017), 565 (Yang et al., 2018), 1,179 (Alexander et al., 2018), and 3,186 (Weng et al., 2018) diabetes-related genes, respectively. However, only 7.82% (392 of 5,012 genes) of these genes were simultaneously identified in multiple EWASs.

Another example is the identification of Parkinson’s disease–related DNA methylation probes based on three independent EWASs, corresponding to 194 genes (Moore et al., 2014; Chuang et al., 2017; Kaut et al., 2017). Unfortunately, the intersection of only one gene, STK38L, existed in these three studies. Therefore, traditional probe-based EWASs have some limitations in identifying phenotype-related genes based on differential probes. Moreover, how to integrate DNA methylation values of different probes on the same gene and characterize the DNA methylation degree at the gene level has become a challenge to traditional EWASs.

**FIGURE 1:**
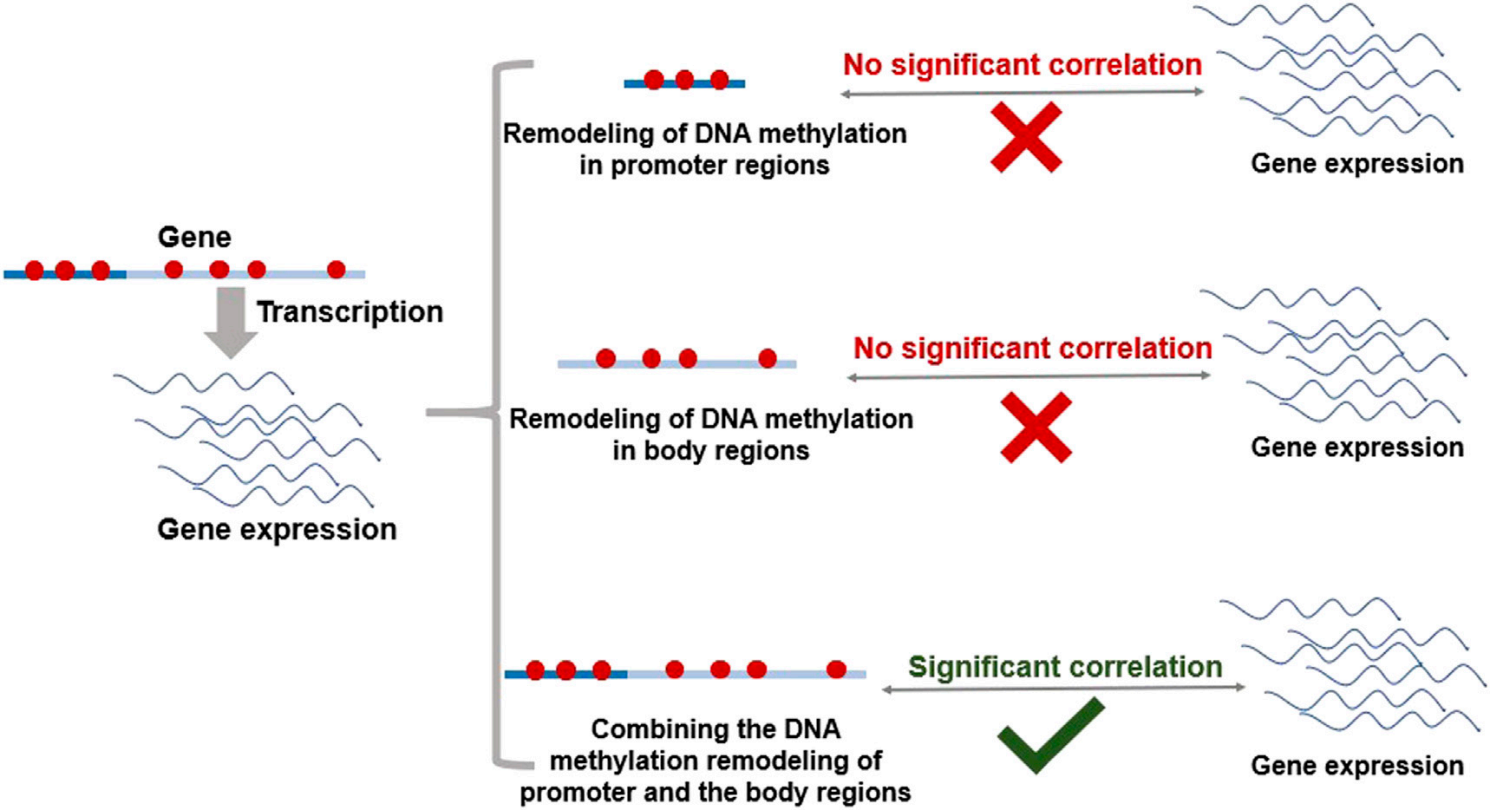
Correlations of DNA methylation remodeling in promoter and body regions with gene expression. Dark blue lines represent the promoter regions of the gene, and light blue lines represent the body regions of the gene. Red dots represent DNA methylation sites on the genome.

Because methylation remodeling has a certain degree of randomness and complexity on the genome, there is no significant correlation to only consider the remodeling of DNA methylation in promoter regions or to only consider the remodeling of DNA methylation in body regions with gene expression (Figure 1). Therefore, this study proposed that, by combining the DNA methylation remodeling of promoter regions and body regions, we could identify robust methylation associations with the phenotype at gene level (Figure 1). According to a recent study, there is a significant positive correlation between the methylation of gene body difference to promoter (MeGDP) and gene expression of FPKM (Fragments Per Kilobase of exon per Million fragments mapped) in variety of cell lines, which was detected by whole-genome DNA methylation method of Guide Positioning Sequencing (GPS) (Li et al., 2019a). In liver and hepatoma cell line 97L, the correlation coefficient is as high as 0.67 (*p*-value < 2.2e–16) (Li et al., 2019a). This result is consistent with our conjecture and suggests that DNA methylation differences between the gene promoter and body regions can be used as a DNA methylation index to predict gene expression (Figure 1).

Based on the above correlation, this study proposed the statistical difference of DNA Methylation between Promoter and Other Body Region (SIMPO) algorithm to mine gene-level DNA methylation associations with phenotype. It showed the robustness of SIMPO-identified differential genes in the same dataset and between different datasets through three smoking phenotype-related DNA methylation datasets. The results also showed that the biological efficacy of SIMPO-identified differential genes is comparable to those predicted by traditional probe-based methods. In addition, we further applied the SIMPO algorithm to predict insulin resistance (IR)– and Parkinson’s disease (PD)–associated genes and revealed the biological significance of corresponding genes.

## Materials and Methods

### Collection of DNA Methylation and Transcription Data

First, this study collected transcription and DNA methylation data from the MESA (Multi-Ethnic Study of Atherosclerosis) Epigenomics and Transcriptomics Study. This study has been launched to investigate potential gene expression regulatory methylation sites in humans by examining the association between CpG methylation and gene expression in purified human monocytes from 1,202 individuals (ranging 44∼83 years of age) and proved that blood monocyte transcriptome and epigenome can reveal loci associated with human age (Reynolds et al., 2014). We downloaded the above data from the NCBI GEO (Gene Expression Omnibus) database (GEO accession: GSE56045 and GSE56046).

Next, we used three smoking phenotype-related DNA methylation datasets to test the robustness of the SIMPO algorithm. Previous studies have found that smoking is associated with a variety of diseases by affecting DNA methylation and causing abnormal gene expression (Dogan et al., 2014; Tsaprouni et al., 2014; Su et al., 2016). For example, based on peripheral blood DNA methylation data of 464 individuals who were current, former, and never smokers (GEO accession: GSE50660), researchers have identified 15 methylation sites associated with smoking (Tsaprouni et al., 2014). In addition, the GSE53045 dataset contains DNA methylation data extracted from the peripheral mononuclear cell of 50 smokers and 61 nonsmokers. Moreover, 910 significant loci have been predicted after Benjamini-Hochberg correction based on this dataset (Dogan et al., 2014). The third smoking phenotype-related DNA methylation dataset was collected from GSE85210. This dataset included DNA methylation data of blood cells from 172 smokers and 81 nonsmokers and revealed that 738 CpGs were significantly associated with current smoking (Su et al., 2016).

Data of IR-related DNA methylation BeadChip analyzed in this study were also downloaded from the NCBI GEO database (GEO accession: GSE115278). This dataset uses Illumina HumanMethylation450 BeadChip’s GPL16304 platform and contains DNA methylation data of peripheral white blood cells collected from 74 HOMA-IR (i.e., homeostasis model assessment of insulin resistance) >3, and 258 HOMA-IR ≤ 3 individuals. Furthermore, based on this data, a rigorous statistical analysis revealed that 478 CpGs showed a differential methylation pattern between individuals with HOMA-IR ≤ 3 and >3 (Arpón et al., 2019).

Two PD-related DNA methylation datasets were downloaded from the NCBI GEO database (GEO accession: GSE72774 and GSE111629). These dataset use Illumina HumanMethylation450 BeadChip’s GPL13534 platform. GSE72774 contains DNA methylation data of whole blood collected from 289 individuals with PD and 219 control samples; then, these researchers obtained 82 genome-wide significant CpGs of PD (Chuang et al., 2017). Whole blood DNA methylation data of GSE111629 were collected from 335 PD individuals and 237 controls.

### Prediction of Phenotype-Associated Genes Based on SIMPO Algorithm

Previous research found that the DNA methylation difference between the promoter region and the body region is highly related to the expression level of the gene (Zeilinger et al., 2013). The input data of the SIMPO algorithm are the DNA methylation beta value of cg probes that are located in the promoter regions (including TSS200 or/and TSS1500) and the other regions (including gene body, 3′UTR, 5′UTR, and 1stExon) (Table 1). The statistical difference method *t*-test is used in SIMPO, and the degree of difference (SIMPO score) is used to characterize the DNA methylation remodeling of corresponding genes:

$$SimPo\;score=\frac{\bar{\text{x}}-\bar{\text{y}}}{{\text{S}}_{\text{w}}\sqrt{\left(1/m\right)+\left(1/n\right)}}\;∼t\left(m+n-2\right),$$

where

$${\text{S}}_{\text{w}}^{2}=\frac{1}{\text{m}+\text{n}-2}\left[\left(\text{m}-1\right){\text{S}}_{1}^{2}+\;\left(\text{n}-1\right){\text{S}}_{2}^{2}\right].$$

**TABLE 1:** The description of the SIMPO algorithm and the traditional method.

Abbreviation	Description
SIMPO-TSS200	Using the TSS200 probe as the promoter and the other probes as the other regions (including gene body, 3′UTR, 5′UTR, 1stExon, TSS1500)
SIMOP-TSS1500	Using the TSS1500 probe as the promoter region and the other probes as the other regions (including gene body, 3′UTR, 5′UTR, 1stExon, TSS200)
SIMOP-TSS200&TSS1500	Using the TSS200 and TSS1500 probes as promoter regions and the other probes as other regions (including gene body, 3′UTR, 5′UTR, 1stExon)
DMPs	The traditional EWAS algorithm calculates differentially methylated positions between the phenotypic group and the control group through the R package "minfi"
DMGs	The traditional EWAS algorithm is based on DMPs mapping to correspond to differentially methylated genes

Here, $\bar{\text{x}}$ is the average DNA methylation value of all probes that are located in the other region (including gene body, 3′UTR, 5′UTR, 1stExon); $\bar{\text{y}}$ is the average DNA methylation value of all probes that are located in the promoter region; m is the number of probes that are located in the other region (including gene body, 3′UTR, 5′UTR, 1stExon); n is the number of probes that are located in the promoter region; ${\text{S}}_{1}^{2}$ is the variance of DNA methylation values of probes that are located in the other region (including gene body, 3′UTR, 5′UTR, 1stExon); ${\text{S}}_{2}^{2}$ is the variance of DNA methylation values of probes that are located in the promoter region. In addition, since SIMPO algorithm is derived from *t*-test and the SIMPO score relates to the number of probes, in order to ensure the reliability of the SIMPO score, we only selected genes with other region-located and promoter region-located probes greater than or equal to five for further calculation.

**FIGURE 2:**
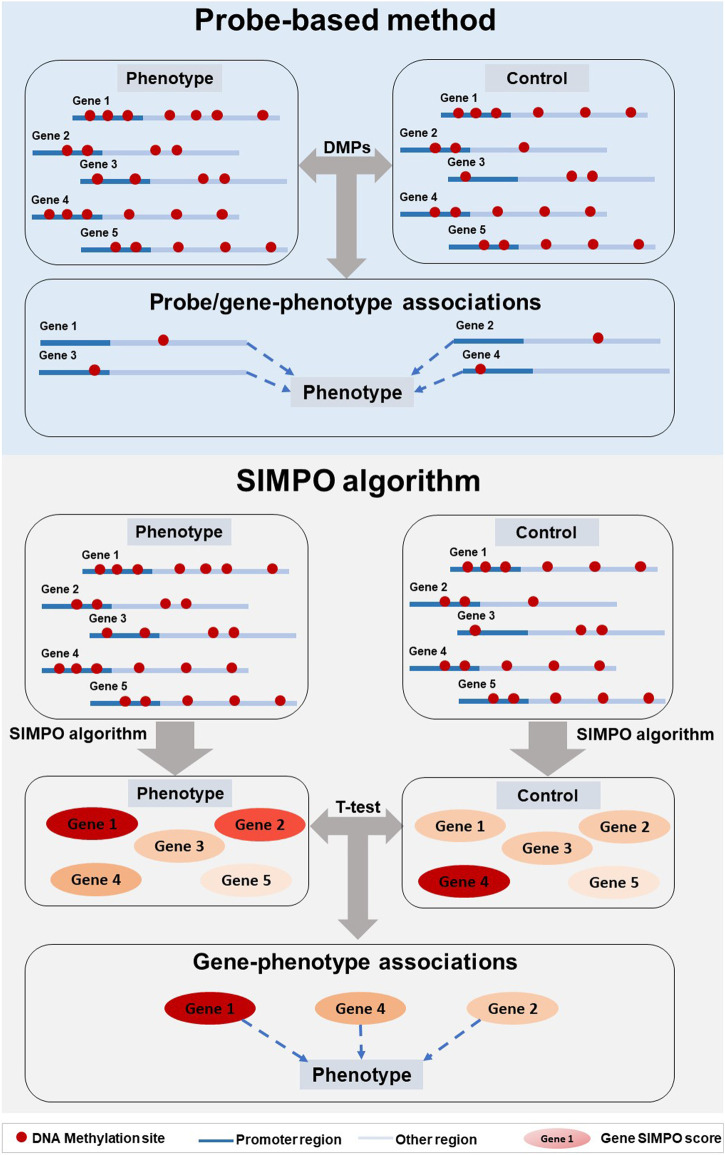
Pipeline comparison of probe-based method and SIMPO algorithm.

Based on the SIMPO algorithm, this study separately calculated the SIMPO score of each gene in the above seven DNA methylation data (GSE56046, GSE50660, GSE53045, GSE85210, GSE115278, GSE72774, and GSE111629). Next, we calculated the differences of gene SIMPO scores between the phenotypic individuals and control groups based on the adjusted *t-test* (Figure 2). In this study, we used the commonly accepted 0.05 as the threshold of *p*-value. When the *p*-value calculated by adjusted *t-test* (between the phenotypic individuals and control groups) of a certain gene is less than 0.05, we predict that this gene is significantly associated with the corresponding phenotype (Figure 2). Through the above calculations, we will obtain genes that are significantly related to multiple phenotypes (involving smoking, IR and PD) in terms of DNA methylation.

### Collection of Known Disease-Associated Genes

In this study, known disease-associated genes were collected from the DisGeNET database (http://www.disgenet.org) and the SCG-Drug database (http://zhanglab.hzau.edu.cn/scgdrug) (Piñero et al., 2017; Quan et al., 2019). DisGeNET database integrates multiple disease gene databases and gene-disease associations (GADs) reported in a large number of works of literature. Data sources include UniProt, Comparative Toxicogenomics Database (CTD), ClinVar, Orphanet, GWAS Catalog, and Genetic Association Database. The latest version is v5.0, which contains 561,119 gene-disease pairs involving 17,074 genes and 20,370 diseases. In addition, DisGeNET v5.0 has developed a gene-disease relationship scoring model with scores between 0 and 1. Higher scores indicate higher confidence in the gene-disease relationship (Piñero et al., 2017). DisGeNET score for the gene-disease relationship is supported by multiple pieces of evidence and has high confidence. The SCG-Drug database collects gene-disease associations from multiple sources (Quan et al., 2019). Similar to DisGeNET, SCG-Drug also annotates the scoring model of gene-disease associations.

### Noise Generation

In order to verify the robustness of SIMPO algorithm in the same dataset, this study added random noise of 0.1–1° to the DNA methylation beta value of each probe. Firstly, because the range of normalized DNA methylation beta is −1 to 1, we generated the random numbers in the range of −1 to 1 through the Python random module. Next, we multiplied the random number by 0.1–1 and obtained the random noise values of about 0.1–1. Third, we added random noise values to the original DNA methylation beta values and received the new beta values. We further used these new DNA methylation beta values of probes in the SIMPO score calculation.

### KEGG Pathway Enrichment

We enriched the KEGG pathway of PD-associated differential genes through GSEA (Gene Set Enrichment Analysis) (Subramanian et al., 2005). The rank of differential genes was derived from *p*-values of *t*-test based on SIMPO scores, and KEGG pathway gene sets were downloaded from the Molecular Signatures Database (MSigDb, c2.cp.kegg.v6.2.symbols.gmt). GSEA calculations are performed based on the R packages of “dplyr” and “GSEABase.” In addition, we performed the KEGG pathway enrichment analyses for the IR-associated gene sets by using the Enrichr database (https://amp.pharm.mssm.edu/Enrichr/).

## Results

### Correlation Between SIMPO Score and Transcription Value of Gene

The DNA methylation feature (SIMPO score) of each gene was extracted based on the SIMPO algorithm, and the Spearman correlation test was used to test the correlation between the SIMPO score and mRNA transcription average of each gene in 1,202 samples (GSE56045 and GSE56046 datasets). In this study, we used a commonly accepted *p*-value of 0.05 as the threshold for determining the significant correlation between DNA methylation and mRNA transcription. When the *p*-value of Spearman correlation test is less than or equal to 0.05, we think the SIMPO scores of genes are significantly related to the average mRNA transcription. The results are shown in Supplementary Figure S1: for the SIMPO-TSS200 algorithm, the SIMPO scores of 43.44% of the genes are significantly related to the average mRNA transcription (Supplementary Figure S1G) (Supplementary Table S1); for the SIMPO-TSS1500 algorithm, the SIMPO scores of 41.22% of the genes are significantly related to the average mRNA transcription (Supplementary Figure S1H) (Supplementary Table S2); for the SIMPO-TSS200&TSS1500 algorithm, the SIMPO scores of 41.18% genes are significantly correlated with the average mRNA transcription (Supplementary Figure S1I) (Supplementary Table S3). The above results are similar to the significant correlation ratio of probes based on DNA methylation beta value (Supplementary Figures S1A–F). It is indicated that the SIMPO score of the gene has a good correlation with the average mRNA transcription, and the SIMPO score can contain the original DNA methylation information of the gene.

### Robustness Verification of SIMPO Algorithm

Based on the SIMPO algorithm and traditional probe-based algorithm, DNA methylation features of different genes of smokers and healthy people were obtained. Then, the significantly associated probes/genes of smoking were predicted through differential analysis (calculated by *t*-test). The numbers of differential genes (*p*-value ≤ 0.05) obtained from these three smoking DNA methylation datasets are shown in Supplementary Figure S2 (Supplementary Tables S4–S8).

For a particular gene, multiple probes contained in it will get different *p*-values. We selected the max *p*-value and the min *p*-value of the probe to represent the correlation between this gene and the smoking phenotype and then obtained the ranking of these genes, respectively. We compared the intersection of the top N genes to show the robustness of traditional EWAS, which often focus only on the DNA methylation level of the probes for the same dataset. The results are shown in Supplementary Figure S3. For the probe-focused study, the robustness in the same dataset is weak, and only about 8% of the genes have intersections.

Next, in order to test the robustness of the SIMPO algorithm in the same dataset, this study added random noise of 0.1–1° to the three DNA methylation data related to the smoking phenotype. Moreover, the intersections of top N smoking-associated genes identified by the original data and after adding noise-data between the traditional probe-based methods (DMPs and DMGs) and the SIMPO algorithm were compared. The results are shown in Supplementary Figures S4,S5. For the GSE50660 and GSE85210 datasets, when different levels of noise are added, the gene intersections obtained by the SIMPO algorithm were more significant than probe-based methods. Hence, the robustness of SIMPO is better than the traditional probe-based method for the same dataset.

**FIGURE 3:**
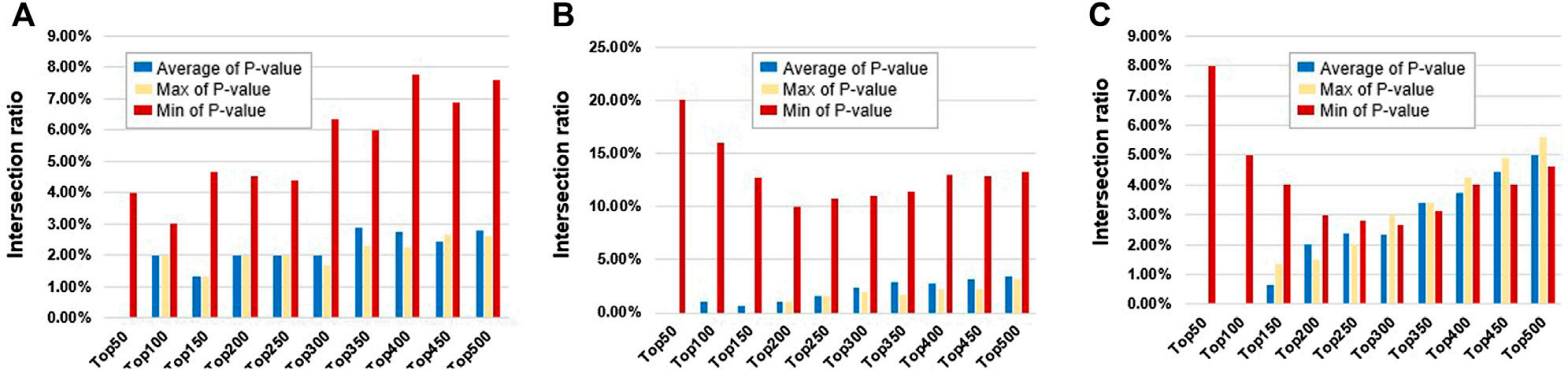
Intersection ratios of top N differential genes derived from different probes between different datasets (between the average, max, and min *p*-value of probes). The blue bars represent top N genes derived from the average *p*-value of probes, the yellow bars represent top N genes derived from max *p*-value of probes, and the red bars represent top N genes derived from min *p*-value of probes. **(A)** Between GSE50660 and GSE53045 dataset. **(B)** Between GSE50660 and GSE85210 dataset. **(C)** Between GSE53045 and GSE85210 dataset.

For traditional probe-based methods, because the same gene often contains multiple methylation probes, the same gene will get multiple smoking-related *p*-values. For the same gene, this study intended to select the average, the max, and the min *p*-value to represent the correlation between this gene and the smoking phenotype. The intersections of top N genes between dataset pairs were then used to show the robustness of the traditional method between different datasets. The results are shown in Figure 3. The min *p*-value probe-selected method is the most robust among different datasets. However, the proportion of intersections is relatively small at only about 5%.

**FIGURE 4:**
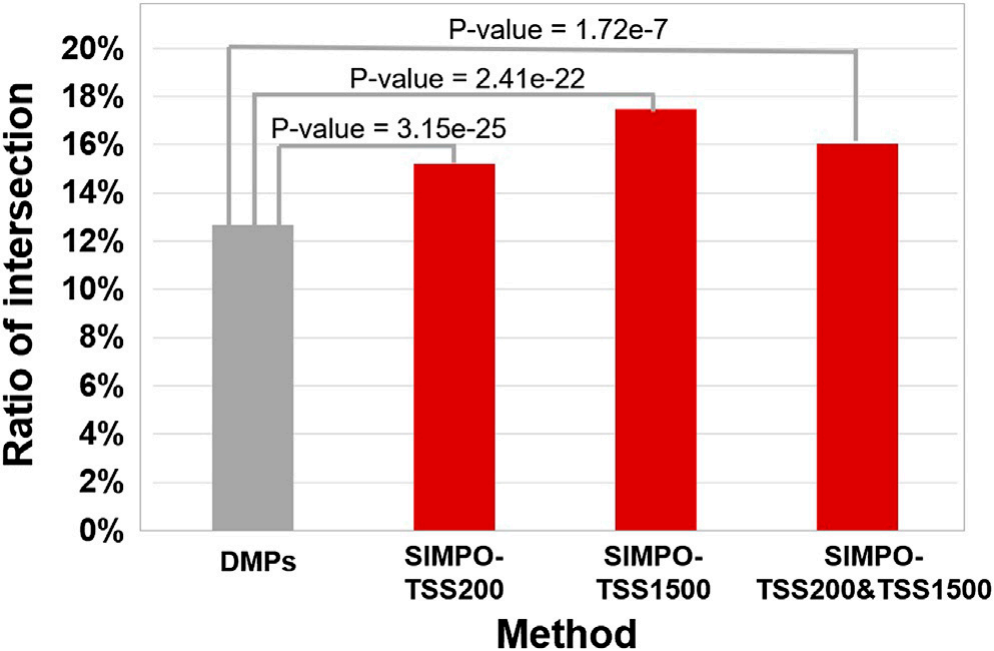
The comparison of the intersection ratio of differential genes/probes derived from at least two smoking-related datasets identified by different methods. The *p*-values were calculated by chi-square test.

**FIGURE 5:**
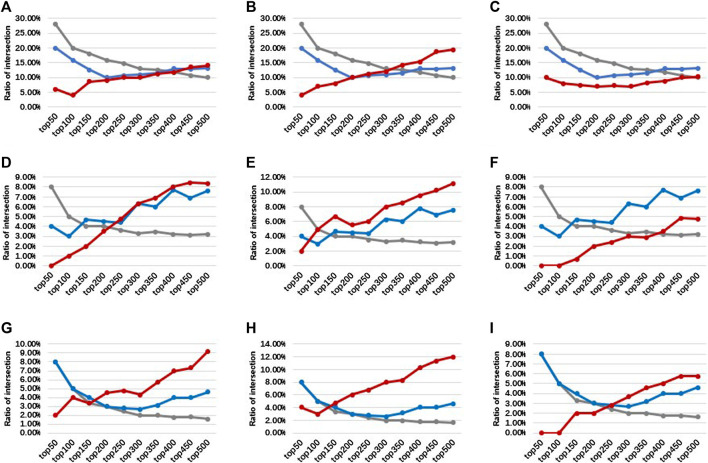
The intersection ratios of top N differential genes/probes between three smoking-related datasets. The gray, blue, and red lines represent the DMPs, DMGs, and SIMPO algorithm methods, respectively. **(A)** For SIMPO-TSS200 algorithm-identified differential genes between GSE50660 and GSE85210 dataset. **(B)** For SIMPO-TSS1500 algorithm-identified differential genes between GSE50660 and GSE85210 dataset. **(C)** For SIMPO-TSS200&1,500 algorithm-identified differential genes between GSE50660 and GSE85210 dataset. **(D)** For SIMPO-TSS200 algorithm-identified differential genes between GSE50660 and GSE53045 dataset. **(E)** For SIMPO-TSS1500 algorithm-identified differential genes between GSE50660 and GSE53045 dataset. **(F)** For SIMPO-TSS200&1,500 algorithm-identified differential genes between GSE50660 and GSE53045 dataset. **(G)** For SIMPO-TSS200 algorithm-identified differential genes between GSE53045 and GSE85210 dataset. **(H)** For SIMPO-TSS1500 algorithm-identified differential genes between GSE53045 and GSE85210 dataset. **(I)** For SIMPO-TSS200&1,500 algorithm-identified differential genes between GSE53045 and GSE85210 dataset.

Comparing the intersection ratio of differential genes/probes derived from at least two smoking-related datasets identified by DMGs and SIMPO showed that the robustness of the SIMPO algorithm (including SIMPO-TSS200, SIMPO-TSS1500, SIMPO-TSS200, and TSS1500) was significantly due to traditional EWAS (Figure 4). In the analysis of the Top N smoking phenotype-related genes, the SIMPO algorithm also obtained better results than the traditional probe-based method (DMPs) as the number of genes increased (Figure 5). In other words, the intersection ratios of smoking-associated genes identified by SIMPO in the two datasets were significantly higher than the DMPs.

### Biological Significance Verification of SIMPO Algorithm

In this study, we verified the biological significance of the SIMPO algorithm by comparing the intersection of known tobacco use disorder-related genes (obtained from SCG-Drug database) with smoking phenotype-related genes that were identified by the traditional probe-based method (DMGs) and the SIMPO algorithm. The results are shown in Table 2. For example, for the GSE50660 dataset, the SIMPO-TSS200 algorithm can calculate the association degrees of 4,782 genes and smoking phenotypes. If these 4,782 genes are used as the background gene set, SIMPO-TSS200 can identify 827 genes that may be significantly associated with the smoking phenotype (*p*-value ≤ 0.05). Among them, 168 (20.31%) genes are known tobacco use disorder-related genes. Based on the same background gene sets (4,782 genes), DMGs method can identify 4,018 significantly associated genes, of which 19.59% (787) are known tobacco use disorder-related genes, slightly lower than SIMPO-TSS200. Similarly, based on the same background gene sets of the corresponding SIMPO algorithm, the proportions of known tobacco use disorder-related genes obtained by the SIMPO-TSS200 and TSS1500 (23.37%) algorithms are higher than DMGs (21.86%) for the GSE50660 dataset; the proportions of these three SIMPO algorithms (19.51% for SIMPO-TSS200, 23.56% for TSS1500, and 22.16% for TSS200 and 1500) are higher than DMGs (18.15%, 22.13%, 20.87%, respectively) for the GSE53045 dataset; the proportions of SIMPO-TSS200 algorithm (19.62%) are higher than DMGs (18.62%) for the GSE85210 dataset (Supplementary Tables S5–S8). In summary, the biological significance of phenotype-related genes identified by SIMPO algorithm is comparable to that of the traditional probe-based method (DMGs).

**TABLE 2:** Ratios of known tobacco use disorder-related genes.

Dataset	SIMPO algorithm	Background gene number	Ratio (DMGs-identified genes)^a^	Ratio (SIMPO-identified genes)^b^
GSE50660	TSS200	4,782	19.59% (787/4,018)	20.31% (168/827)
	TSS1500	4,640	23.07% (955/4,139)	22.13% (156/705)
	TSS200 and 1500	10,893	21.86% (2,050/9,379)	23.37% (383/1,639)
GSE53045	TSS200	4,868	18.15% (868/4783)	19.51% (454/2,327)
	TSS1500	4,697	22.13% (1,027/4,640)	23.56% (551/2,339)
	TSS200 and 1500	10,974	20.87% (2,244/10,752)	22.16% (1,208/5,451)
GSE85210	TSS200	5,794	18.62% (999/5,364)	19.62% (155/790)
	TSS1500	5,368	22.67% (1,153/5086)	22.26% (225/1,011)
	TSS200 and 1500	12,066	20.95% (2,348/1,1206)	20.41% (405/1,984)

^a^ Ratios of DMGs-identified smoking phenotype-related genes (p-value ≤ 0.05).

^b^ Ratios of SIMPO-identified smoking phenotype-related genes (p-value ≤ 0.05).

In the above analyses, we analyzed a set of samples (including 1,202 individuals) that contained both transcriptome and DNA methylation data and showed that the SIMPO scores of ∼40% of genes were significantly correlated with mRNA expression values, proving that SIMPO scores and mRNA expression of genes have good correlations. Next, we used three smoking-related DNA methylation datasets to validate the robustness of the SIMPO algorithm. The results showed that the robustness of the SIMPO is significantly better than the traditional probe-based methods for the same datasets and between different datasets. Finally, by comparing with known tobacco use disorder-associated genes, it is proved that the biological significance of phenotype-related genes identified by SIMPO algorithm is comparable to that of the traditional probe-based methods. Therefore, we will use SIMPO-TSS1500 as a representative of SIMPO algorithm for the following analyses. In summary, the SIMPO algorithm has good robustness and biological efficacy and can be further applied to phenotype or disease research in the field of epigenetic biology.

### Application of SIMPO Algorithm in Insulin Resistance–Associated Gene Prediction

In this study, the SIMPO-TSS1500 algorithm was used to mine gene-level methylation remodeling pattern for IR-related dataset (GEO accession: GSE115278), and then *t-*test was applied to identify differential genes between individuals with HOMA-IR ≤ 3 and > 3. As a result, 990 IR-associated genes were predicted by SIMPO-TSS1500 (Supplementary Table S9). On the other hand, starting from the same dataset, another study has identified a total of 478 CpGs based on the traditional method, covering 499 differential genes (Arpón et al., 2019). Because IR is a pathological condition in which cells fail to respond appropriately to insulin, and it is a hallmark of type 2 diabetes (Schinner et al., 2005; Arpón et al., 2019), we speculated that the above IR-related differential genes are associated with diabetes. By querying the known diabetes-associated genes recorded in the SCG-Drug database, it was found that only 77 genes of the 499 genes (15.43%) identified by traditional methods were known as diabetes-associated genes. For the 990 genes identified by the SIMPO-TSS1500 algorithm, the ratio is 44.44% (440 of 990 genes) (Supplementary Table S9), which is significantly higher than the traditional method (*p*-value = 1.20e–28, based on chi-square test).

Then, according to the *p*-values of the differential genes obtained by the *t*-test, from small *p*-value (most significant) to large *p*-value (least significant), we obtained the top 100 ∼ top 1,000 gene sets related to IR. Similarly, through the probe-based method, we also obtained the top 100 ∼ top 1,000 gene sets. It is worth reminding that when a gene corresponds to multiple probes, we use the probe with the smallest *p*-value to represent this gene and to rank. Based on the KEGG pathway enrichment of the Enrichr database, the results showed that multiple top N gene sets identified by SIMPO-TSS1500 were enriched in diabetes-related KEGG pathways (Table 3), while the top N gene sets identified by probe-based methods were not enriched in corresponding pathways.

**TABLE 3:** The diabetes-related KEGG pathway enrichment of top N gene sets calculated by SIMPO-TSS1500.

Diabetes-related KEGG pathway	Enriched gene set
Cell cycle	Top900; Top1000
Maturity onset diabetes of the young	Top700; Top800; Top900; Top1000
Neurotrophin signaling pathway	Top600
P53 signaling pathway	Top300; Top400; Top500; Top600; Top700; Top800; Top900; Top1000
Wnt signaling pathway	Top100; Top200; Top300; Top400; Top500; Top800; Top900; Top1000

^a^ Obtained from Enrichr database (http://amp.pharm.mssm.edu/Enrichr/).

**FIGURE 6:**
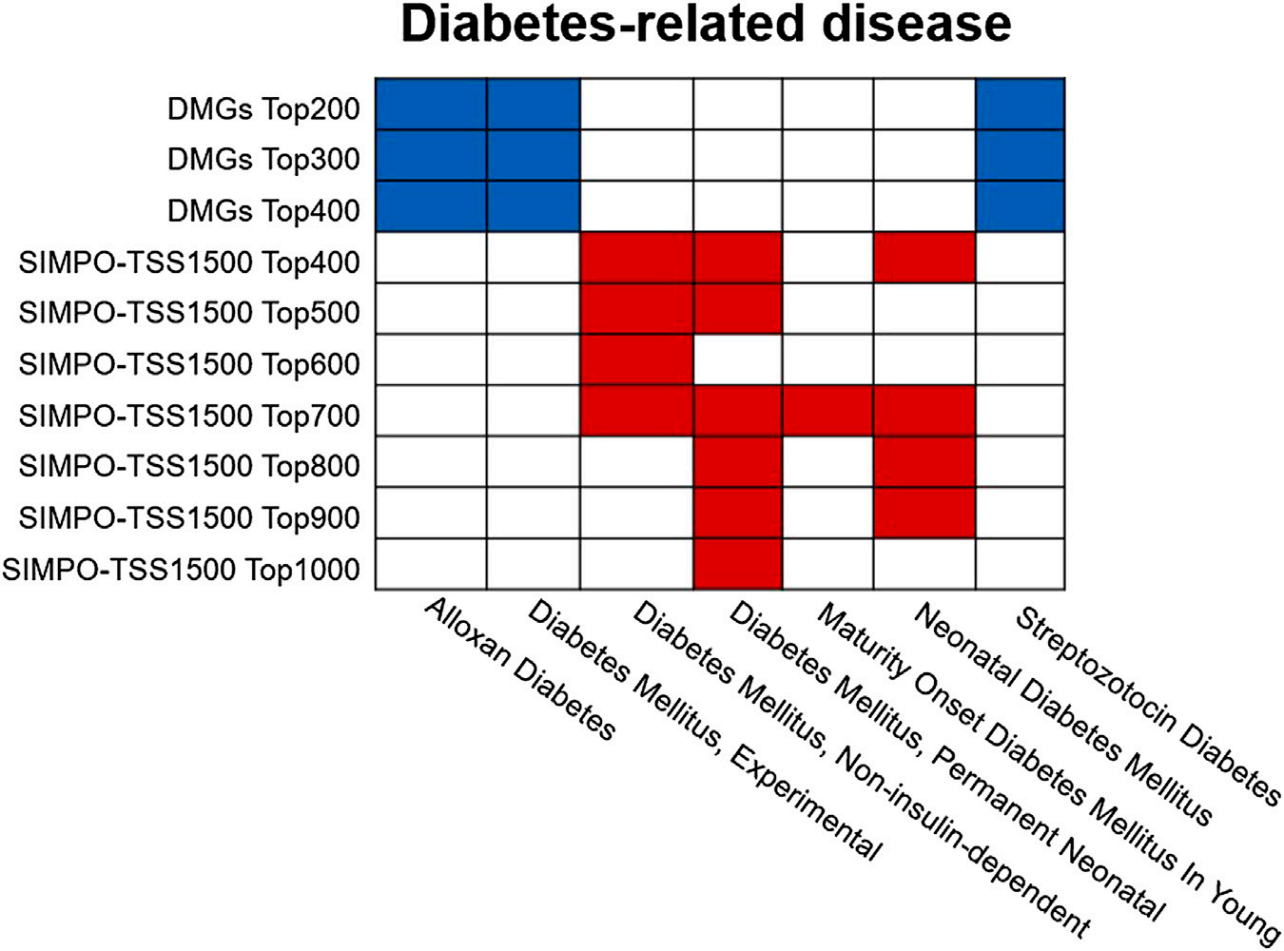
The diabetes-related disease enrichment of DMGs- and SIMPO-TSS1500-predicted top N gene sets. Enrichment analysis was obtained from the Enrichr database (http://amp.pharm.mssm.edu/Enrichr/). The diabetes-related diseases include alloxan diabetes, experimental diabetes mellitus, non-insulin-dependent diabetes mellitus, permanent neonatal diabetes mellitus, maturity onset diabetes mellitus in young, neonatal diabetes mellitus, and streptozotocin diabetes. The blue squares indicate that the DMGs-predicted top N gene set is significantly associated with the corresponding diabetes-associated disease; the red squares indicate that the SIMPO-TSS1500-predicted top N gene set is significantly associated with the corresponding diabetes-related disease.

In addition, we also conducted disease enrichment for the IR-associated gene sets predicted by SIMPO-TSS1500 and DMGs-based methods. The results are shown in Figure 6. SIMPO-TSS1500-predicted top N gene sets were enriched to a variety of diabetes-related diseases through Enrichr database, including non-insulin-dependent diabetes mellitus, permanent neonatal diabetes mellitus, maturity onset diabetes mellitus in young, and neonatal diabetes mellitus, and obtained 15 gene sets–disease associations. However, DMGs-predicted top N gene sets only obtained nine such associations. In summary, the results show that the biological significance of IR-associated genes predicted by SIMPO-TSS1500 is better than those predicted by DMGs-based methods.

### Application of SIMPO Algorithm in Parkinson’s Disease–Associated Gene Prediction

SIMPO-TSS1500 algorithm was further used in mining gene-level methylation remodeling of PD patients and control individuals. Then, 959 significant differential genes for the GSE72774 dataset (Supplementary Table S10) and 1,077 significant differential genes for the GSE111629 dataset related to PD have been identified by *t*-test (Supplementary Table S10). In addition, combining the above two DNA methylation datasets, previous researchers predicted a total of 82 PD-related significant difference CpGs based on the traditional EWAS method, corresponding to 62 genes (Chuang et al., 2017). By querying the known PD-associated genes in SCG-Drug database, it was found that only four of 62 genes (6.45%) identified by the traditional method were known PD-associated genes. For the SIMPO-TSS1500-identified PD-associated genes, the ratios were 9.19% (for GSE111629) and 9.28% (for GSE72774) (Supplementary Table S10), which are higher than the traditional methods.

Then, this study enriched the KEGG pathway for SIMPO-TSS1500-predicted differential gene sets of PDs through GSEA. The results are shown in Table 4. These two PD-related gene sets were enriched to 12 KEGG pathways. By querying the biological function annotations for the pathways on the KEGG website (https://www.genome.jp/kegg/pathway.html), it was found that four pathways are related to nervous system diseases, including Alzheimer’s disease, Inositol phosphate metabolism, phosphatidylinositol signaling system, and purine metabolism.

**TABLE 4:** The KEGG pathway enrichment of PD-associated genes calculated by SIMPO-TSS1500.

Dataset	KEGG pathway	Annotation
GSE72774	Alzheimer’s disease	Nervous system diseases
GSE72774	Cysteine and methionine metabolism	\
GSE72774, GSE111629	Cytokine cytokine receptor interaction	\
GSE111629	Glycolysis gluconeogenesis	\
GSE72774, GSE111629	Gnrh signaling pathway	\
GSE72774, GSE111629	Inositol phosphate metabolism	Nervous system diseases
GSE72774	Jak stat signaling pathway	\
GSE111629	Lysosome	\
GSE111629	Phosphatidylinositol signaling system	Nervous system diseases
GSE72774	Purine metabolism	Nervous system diseases
GSE72774	Snare interactions in vesicular transport	\
GSE111629	Viral myocarditis	\

^1^ Calculated by GSEA (Gene Set Enrichment Analysis).

SCG-Drug and DisGeNET databases collected gene-disease associations from multiple sources, and both annotated the credibility scores of gene-disease associations. This study compared SIMPO-TSS1500-predicted PD-related differential gene sets with the top 10% scored PD pathogenic genes recorded in DisGeNET and SCG-Drug. The intersections of SIMPO-TSS1500-predicted gene sets with known PD-causing genes were significantly higher than the background databases (Table 5) (Supplementary Table S10). The above results further proved the reliability of the SIMPO-predicted PD-associated genes. Moreover, it also reflects the robustness of mining the statistical difference of DNA methylation between the promoter and other regions (SIMPO algorithm) to identify gene-level associations with a given phenotype from the side.

**TABLE 5:** The known PD-associated gene enrichment of SIMPO-TSS1500-calculated genes.

Dataset	Known PD gene source	SIMPO-TSS1500-derived ratio	Background ratio	*p*-value^a^
GSE111629	SCG-Drug^b^	9.19% (99/1,077)	7.21% (351/4,868)	3.26E-03
	DisGeNET^c^	7.15% (77/1,077)	5.88% (286/4,868)	2.79E-02
GSE72774	SCG-Drug^b^	9.28% (89/959)	7.21% (351/4,868)	4.27E-03
	DisGeNET^c^	7.40% (71/959)	5.88% (286/4,868)	1.68E-02

^a^ Calculated by Hypergeometric test.

^b^ Known PD-associated genes were collected from SCG-Drug (http://zhanglab.hzau.edu.cn/scgdrug).

^c^ Known PD-associated genes were collected from DisGeNET (http://www.disgenet.org).

## Discussion

In recent years, through EWAS, researchers have identified thousands of phenotype-related differential methylation sites. However, since the same gene may contain hundreds of methylation sites, the DNA methylation beta values at different sites vary widely. Furthermore, DNA methylation remodeling has a certain degree of randomness on the genome. As a result, for multiple EWASs focused on the same phenotype, the intersections of identified differential methylation site are small, which makes it challenging to identify phenotype-associated genes and analyze epigenetic mechanisms. Therefore, how to integrate the methylation values of different sites on the same gene and identify robust gene-level associations with phenotype becomes a challenge in the epigenetics field.

In this study, by analyzing a set of individual samples containing both transcriptome and DNA methylome data, it was found that the SIMPO scores of ∼40% genes were significantly correlated with transcription of mRNA, demonstrating that the SIMPO scores of genes have a reasonable correlation with gene expression. Then, three DNA methylation datasets related to the smoking phenotype were used to test the robustness of SIMPO algorithm. The results showed that the robustness of the SIMPO algorithm in the same dataset and between different datasets was significantly better than the traditional EWAS method. Finally, through comparing with known tobacco use disorder pathogenic genes, it is proved that the biological significance of phenotype-related genes identified by SIMPO algorithm is comparable to that of the traditional probe-based methods. Next, we further applied the SIMPO-TSS1500 algorithm to predict IR- and PD-associated genes and proved the biological significance of corresponding genes. On the other hand, our research group previously applied the SIMPO algorithm to the prediction of disease-related biomarkers. As a result, these studies successfully identified several DNA methylation biomarkers related to the onset of type 2 diabetes and colorectal cancer and were supported by clinical trials (Quan et al., 2020a; Liang et al., 2020). In summary, SIMPO-TSS1500 algorithm has good robustness and biological significance. Therefore, we recommend that SIMPO-TSS1500 algorithm, which uses the TSS1500 probe as the promoter region and the other probes as the other region (including gene body, 3′UTR, 5′UTR, 1stExon, TSS200), can be further applied to identifying significantly phenotype-related genes in the field of epibiology.

However, the SIMPO algorithm still has some shortcomings. In order to ensure the stability of the SIMPO algorithm, it requires that the promoter region and other regions of a gene contain five or more probes to obtain a SIMPO score. Therefore, a large number of genes containing a small number of probes will be lost. At present, the number of genes that can be calculated by SIMPO-TSS200 and SIMPO-TSS1500 is only about 5,000, and the number of genes that can be calculated by SIMPO-TSS200 and 1500 is only about 10,000. It is much smaller than the number of genes contained in the human genome. As a result, some critical functional genes have been missed for the current SIMPO algorithm. Fortunately, with the popularity of the Illumina 850K BeadChip in EWAS, which contains more than 850,000 probes, the number of genes that the SIMPO algorithm can calculate will increase significantly. In addition, the effectiveness of the SIMPO algorithm is only verified in DNA methylation data of human at this stage, excluding other common model animals (such as mouse, rat, and *drosophila*). Therefore, the exploration of the effectiveness of the SIMPO algorithm in other species will be the focus of our future research. As a supplement of the traditional methods, SIMPO algorithm provides a new insight for assessing the degree of gene methylation. The different analysis methods of methylation chip can help us better understand the gene-level methylation associations with phenotype, providing a different perspective in exploring the biological issues. We believe that the combination of these methods can help us understand the regulation mechanism of gene methylation more deeply and solve scientific problems more effectively.

## Data Availability

The original contributions presented in the study are included in the article/Supplementary Material; further inquiries can be directed to the corresponding author.
