# Supplementary material for: Mining the Selective Remodeling of DNA Methylation in Promoter Regions to Identify Robust Gene-Level Associations With Phenotype
Source: Front Mol Biosci. 2021 Mar 26;8:597513. doi: 10.3389/fmolb.2021.597513 (PMC8034267; doi:10.3389/fmolb.2021.597513)
Supplement: Supplementary file 2 [file table2.docx]

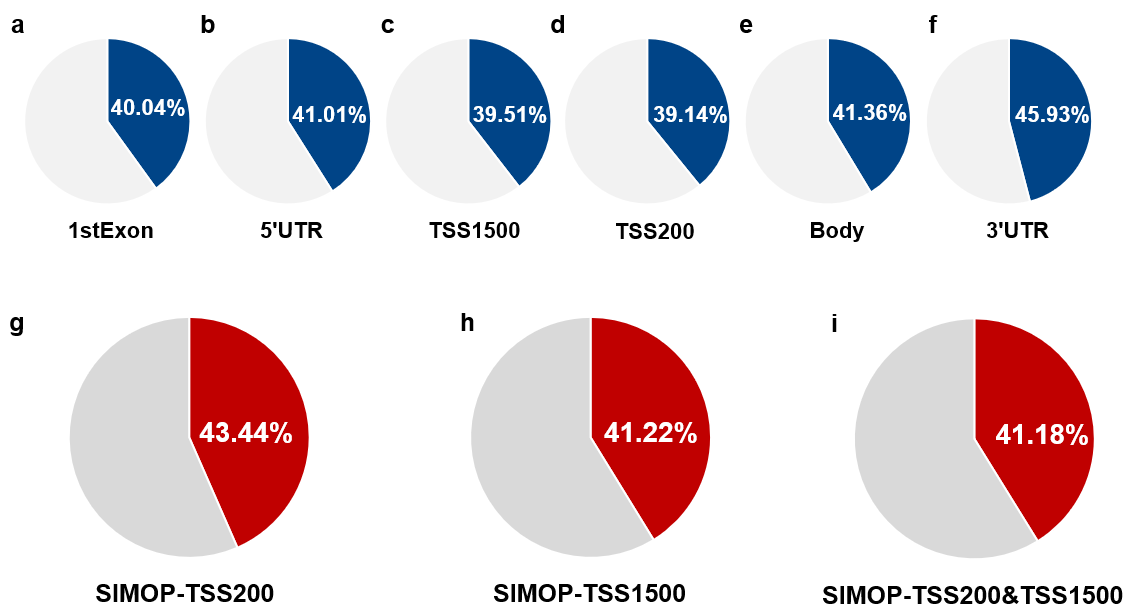


**Supplementary figure 1**. Proportion of probes/genes whose DNA methylation beta values or SIMPO scores are significantly associated with mRNA transcription values. Correlation analysis is based on the Spearman correlation test. (a) for probes located in 1stExon regions. (b) for probes located in 5'UTR regions. (c) for probes located in TSS1500 regions. (d) for probes located in TSS200 regions. (e) for probes located in body regions. (f) for probes located in 3'UTR regions. (g) for SIMPO-TSS200 algorithm-identified genes. (h) for SIMPO-TSS1500 algorithm-identified genes. (i) for SIMPO-TSS200&TSS1500 algorithm-identified genes.


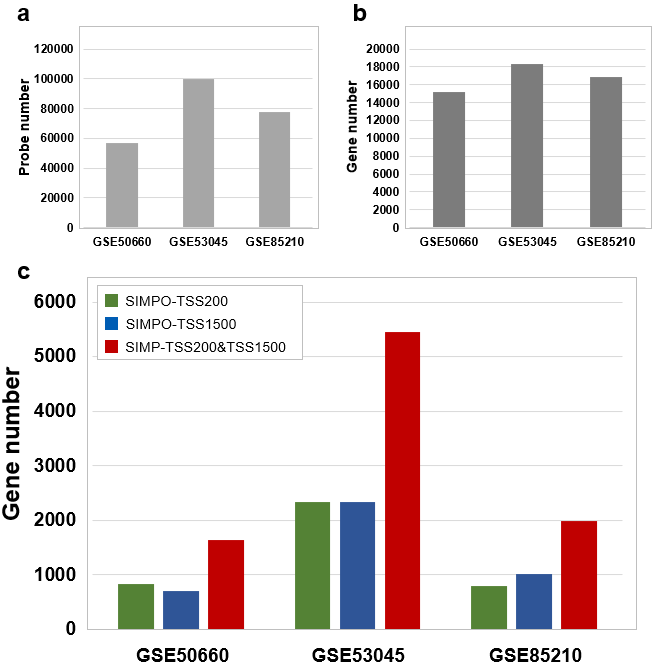


**Supplementary figure 2**. The significantly associated probe/gene numbers of smoking phenotype identified by different methods. (a) The significantly associated probe numbers of three smoking phenotype-related datasets identified by DMPs. (b) The significantly associated gene numbers of three smoking phenotype-related datasets identified by DMGs. (c) The significantly associated probe numbers of three smoking phenotype-related datasets identified by SIMPO algorithms.


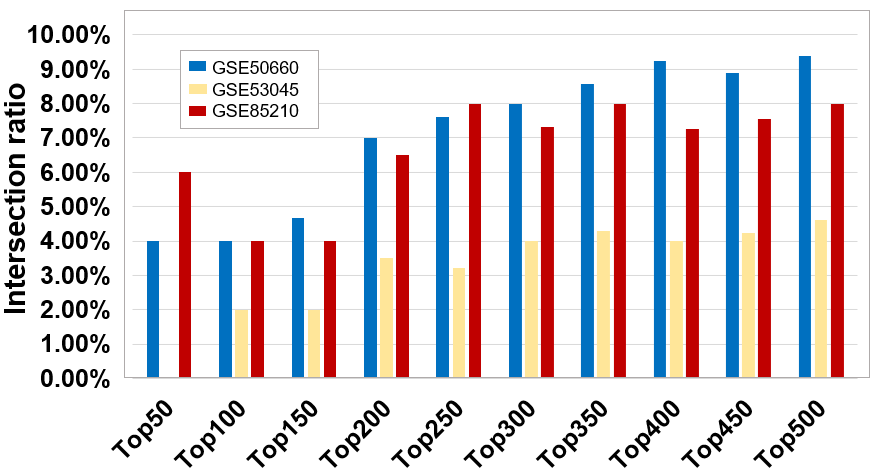


**Supplementary figure 3**. The intersection ratios of top N differential genes derived from a different probe located in the same genes (between the max P-value probe and min P-value probe).


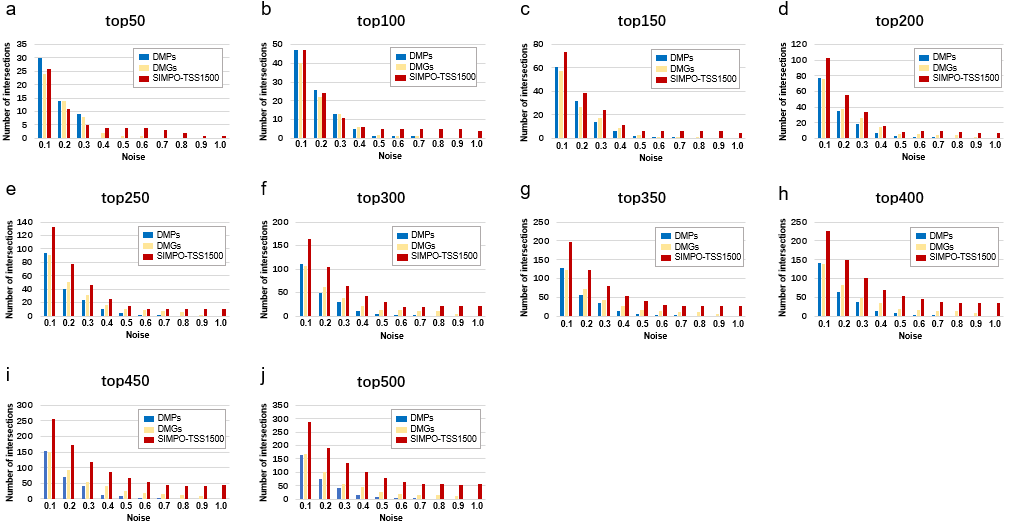


**Supplementary figure 4**. The intersections of top N smoking-associated genes identified by the original data and after adding noise-data of the GSE50660 dataset. The blue bars represent genes identified by the DMPs, the yellow bars represent genes identified by the DMGs, and the red bars represent genes identified by the SIMPO algorithm. (a) for to p50 probes/genes. (b) for top 100 probes/genes. (c) for top 150 probes/genes. (d) for top 200 probes/genes. (e) for top 250 probes/genes. (f) for top 300 probes/genes. (j) for top 350 probes/genes. (h) for top 400 probes/genes. (i) for top 450 probes/genes. (j) for top 500 probes/genes.


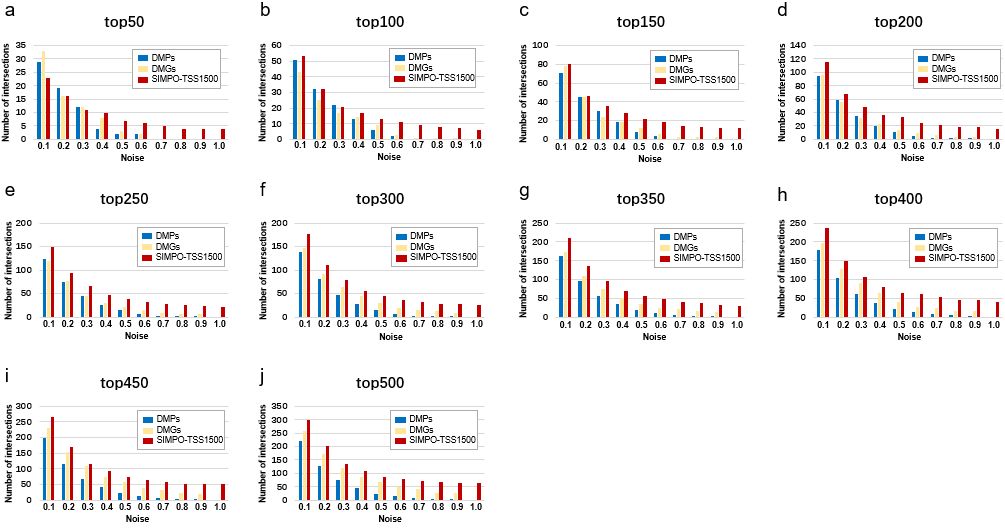


**Supplementary figure 5**. The intersections of top N smoking-associated genes identified by the original data and after adding noise-data of the GSE85210 dataset. The blue bars represent genes identified by the DMPs, the yellow bars represent genes identified by the DMGs, and the red bars represent genes identified by the SIMPO algorithm. (a) for top 50 probes/genes. (b) for top 100 probes/genes. (c) for top 150 probes/genes. (d) for top 200 probes/genes. (e) for top 250 probes/genes. (f) for top 300 probes/genes. (j) for top 350 probes/genes. (h) for top 400 probes/genes. (i) for top 450 probes/genes. (j) for top 500 probes/genes.
